# A Non-Surgical Wearable Option for Bone Conduction Hearing Implants: A Comparative Study with Conventional Bone Conduction Hearing Aids Mounted on Eyeglasses

**DOI:** 10.3390/audiolres14050075

**Published:** 2024-10-11

**Authors:** Federica Di Berardino, Giovanni Ciavarro, Giulia Fumagalli, Claudia Albanese, Enrico Pasanisi, Diego Zanetti, Vincenzo Vincenti

**Affiliations:** 1 Department of Clinical Sciences and Community Health, University of Milan, 20122 Milan, Italy; giuliafumagalli95@gmail.com (G.F.); diego.zanetti@policlinico.mi.it (D.Z.); 2 Audiology Unit, Department of Surgical Sciences, Fondazione IRCCS Ca’ Granda, Ospedale Maggiore Policlinico, 20122 Milan, Italy; 3 Unit of Otorhinolaringology and Otoneurosurgery, Head and Neck Department, University-Hospital of Parma, 43121 Parma, Italy; ciavarrogiovanni@gmail.com (G.C.); enrico.pasanisi@unipr.it (E.P.); vincenzo.vincenti@unipr.it (V.V.); 4 Independent Researcher, 20100 Milan, Italy; claudia.albanese@amplifon.com

**Keywords:** bone conduction implantable device, bone conduction hearing aids

## Abstract

Objectives. This study aimed to compare the audiological benefits of a non-implantable wearable option for a bone conduction (BC) implant mounted on an arch (SoundArc) to those of traditional BC hearing aids (HAs) mounted on eyeglasses (BCHAs) in patients with moderate to severe conductive or mixed hearing loss. Methods: A preliminary cross-sectional observational prospective cohort study was conducted in the Tertiary Audiological Department, University Hospital. Fourteen adults with conductive or mixed hearing loss (PTA at 0.5-1-2-4 KHz = 67 ± 15 dB HL) who had been wearing conventional BCHAs mounted on eyeglasses for at least 3 years and had declined surgical implantation of a bone conduction hearing implant (BCHI) were included in the study. Unaided and aided pure-tone air conduction (AC) and bone conduction (BC) thresholds, as well as speech tests in quiet and noise, were recorded at baseline and in two different settings: with a BCHI mounted on SoundArc^®^ and with their own BCHAs mounted on eyeglasses using two couplers. Participants completed questionnaires in both conditions, including the International Inventory for Hearing Aids (IOI-HA), the Hearing Handicap Inventory for Adults/Elderly (HHIA/E), the Speech, Spatial, and Qualities of Hearing Scale (SSQ), a 10-point visual analog scale (VAS), and the Fatigue Impact Scale (FIS). Results: A significant functional gain was observed in both settings (*p* = 0.0001). Better speech perception in quiet and noise was observed with SoundArc compared to conventional BCHAs on eyeglasses (improvements in word repetition scores in noise: +19.3 at SNR +10 dB, *p* = 0.002; +12.1 at SNR 0 dB, *p* = 0.006; and +11.4 at SNR −10 dB, *p* = 0.002). No significant differences were found in IOI-HA, FIS, and HHIA/E scores. However, significantly better SSQ scores were reported for SoundArc in all domains (*p* = 0.0038). Conclusions: Although patients were accustomed to using BCHAs mounted on eyeglasses, the bone conduction wearable option of the BCHI (SoundArc) proved to be a viable alternative for adult patients with conductive or mixed hearing loss who are unable or unwilling to undergo BCHI surgery.

## 1. Introduction

Patients with conductive or mixed hearing loss can typically benefit from ossiculoplasty or stapedotomy procedures or from bone conduction (BC) or air conduction (AC) behind-the-ear hearing aids. However, surgical rehabilitation can be challenging in patients with active chronic otitis media or after radical mastoidectomy; AC hearing aids are contraindicated in cases of persistent purulent otorrhea, external ear canal atresia, or chronic dermatosis/dermatitis. Revision stapedotomy in otosclerosis patients carries an increased risk of sensorineural damage, and elderly patients with comorbidities may not be fit for surgery [1]. Additionally, some patients refuse to use AC hearing aids for aesthetic reasons. BC hearing aids (BCHAs), usually mounted on eyeglasses, were the conventional solution for this type of hearing loss. Despite their ease of use, BCHAs have shortcomings, including inefficient coupling with the bone, often unsatisfactory results, and poor aesthetic appeal. To address these clinical challenges and achieve better functional outcomes, bone conduction hearing implants (BCHIs) have progressively gained success as reliable and effective rehabilitation tools [1]. The first passive BCHI, a bone-anchored hearing aid (BAHA), was implanted in 1977. It required the surgical implantation of a titanium screw (fixture) that would be osteo-integrated in the temporal squama, with an abutment across the skin (percutaneous), after the elevation of a cutaneous flap, soft tissue reduction, and periosteal incision [2]. Over the last few decades, substantial clinical evidence has supported the audiological efficacy of BCHIs but has also highlighted the need for continued care at the implantation site and the risks of recurrent local infections, soft tissue complications, and implant extrusion [3,4,5]. In response to these issues, several transcutaneous systems have been developed, such as the BAHA Attract^®^ (Cochlear Inc., Molnlycke, Sweden) [6] and the Sophono™ system (Medtronic, Inc., Fridley, MN, USA) [7,8]. Transcutaneous BCHIs are designed to reduce adverse side effects, costs, and the risk of an invasive surgical procedure while also improving visual appeal [9,10,11,12]. However, these devices provide slightly lower audiological benefits compared to percutaneous devices due to the damping effect of soft tissues, as occurs with conventional BCHAs. Even though implant-related soft tissue problems can be resolved with transcutaneous devices, the requirement for surgical positioning still discourages many patients and parents of children from opting for BCHIs. Recently, “active” middle ear implants and transcutaneous BC devices with an implanted actuator (“active” BCHI), such as the Bonebridge^®^ (Medel, Innsbruck, Austria) and the OSIA^®^ (Cochlear co, Lane Cove., Australia), have been developed to amplify bone vibration and improve speech perception abilities [8,13]. When surgery is not possible or contraindicated and amplification by AC hearing aids is not feasible, then implantable hearing devices are the next logical option [1]. However, some patients with conductive or mixed hearing loss still refuse both active and passive BCHIs due to concerns about the invasiveness of the surgery, potential medical complications, and the devices’ actual effectiveness, or because they are unable to receive a BCHI for medical reasons [1,5,14,15,16,17]. In such instances, BC wearable applications of BCHIs, such as softbands or adhesive BCHAs (e.g., Adhear^®^), as described in ref. [15], have been proposed as viable alternatives to surgery [1,15,16].

In our clinical practice, a few patients who were familiar with BCHIs but not candidates for surgery were willing to try wearable solutions such as headbands or adhesive BCHIs, but despite their innovative and powerful design, they rejected these options for aesthetic reasons or because they found them less comfortable to use. Recently, a non-implantable wearable configuration for the BCHI, SoundArc^®^, has been added to the non-surgical options [18]. Its rigid bow, similar to BC headphones used for leisure, aimed to improve the aesthetic appeal to adults while still maintaining a good BC coupling. However, this wearable option was not mentioned in the recent Consensus Statement on Bone Conduction Devices [1]. In the present study, we compared the audiological outcomes, hearing-loss-related fatigue, and residual disability using SoundArc in experienced users of BCHAs mounted on eyeglasses.

## 2. Methods

A prospective, observational, single-subject, repeated-measures study was conducted at a Tertiary Referral University Hospital, with each subject serving as their own control. The study included 14 patients (8 females, 6 males) with bilateral conductive and mixed acquired hearing loss, who were consecutively admitted for consultation. Patients were adults (>18 years) who were experienced BCHA users (having used BCHAs for at least 3 years and preferring to use BCHAs rather than AC hearing aids) and who refused the surgical implantation of a BCHI despite repeated in-depth counseling in accordance with the guidelines. Exclusion criteria were age < 18 years; retro-cochlear or central auditory disorders; cognitive impairment, including mild cognitive impairment or psychiatric conditions; external, middle, and/or inner ear malformations; congenital preverbal hearing loss; and unwillingness to participate in the study.

Patients’ details are listed in Table 1. The median age was 77.8 ± 5.1 years (ranging from 68 to 88 years). Diagnoses included chronic otitis media (12/14 cases, 86%), including 2 patients who had undergone a radical mastoidectomy and otosclerosis (2/14 cases, 14%). This cohort was enrolled for a period of 9 months. Medical history and otoscopy were recorded.

The study protocol included two baseline measurements: (1) unaided pure-tone audiometry [UNI EN ISO 1_tonale]; (2) unaided speech audiometry. Subsequently, three aided free-field thresholds were obtained: (3) aided pure-tone audiometry; (4) aided speech perception tests in quiet; and (5) aided speech perception tests in noise. The air–bone gap (ABG) was measured at 0.25-0.5-1-2-4 KHz by a B71 bone transducer. The speech perception test consisted of the repetition of 10 words presented in an open set (word recognition score, WRS) in both quiet and noise; 4 lists were randomly selected, 1 for each task, among 20 lists of phonetically balanced words of common use and meaningful in the Italian language [19]. Speech perception in noise (cocktail party) was tested with signal-to-noise ratios (SNRs) of +10, 0 dB, and −10 dB HL. All tests were performed in a free-field sound-treated room according to UNI EN ISO 8253-3 at 60 dB HL in both quiet and noise (speech signal at 0° azimuth and noise at 180° azimuth, S0N90) [20]; the loudspeakers were placed 1 m from the center of the participant’s head [21].

All aided tests were performed with the personal BCHAs with which each patient was familiar. In a second round of testing, aided pure-tone and speech audiometry were repeated in a free field using BCHI SoundArc^®^. Seven patients wore a unilateral Baha 5^®^ sound processor, while a more powerful processor (BAHA 5 Power^®^) was needed in 9 out of 14 (64%). All subjects were evaluated after a one-month free trial of SoundArc use. Audiological measurements were performed by the same expert audiologist (GF) to reduce any measurement bias.

All patients were also asked to fill in the following questionnaires regarding the use of their BCHAs: Hearing Handicap Inventory for Adult or Elderly [HHIA/HHIE] [22,23,24,25]; International Inventory for Hearing Aids [IOI—HA] [26]; Speech, Spatial, and Qualities of Hearing Scale [SSQ]; and Fatigue Impact Scale [FIS] [27,28]. The Italian version of the tested questionnaires has already been used in a previous study by the authors [29]. These questionnaires were also collected after the aided trial with SoundArc, together with the collection of a visuo-analogic scale [VAS] graded from 0 (minimum) to 10 (maximum) about wearability (comfort, weight, and aesthetics).

Statistical analysis. The Shapiro–Wilk normality test was first applied to the dataset to assess the normality of continuous variables. A Wilcoxon rank-signed exact test (2-tailed) was used to assess differences between the results obtained with BCHAs and SoundArc. Values were considered statistically significant when *p* < 0.05. All statistics were calculated with the Statistical Package for the Social Sciences 26 for Windows software package (SPSS Inc, Chicago, IL, USA), https://learn.microsoft.com/en-us/windows/package-manager/ (accessed on 1 March 2024).

The study received approval from the Ethical Committee of Fondazione IRCCS Ca’ Granda Ospedale Maggiore Policlinico of Milan, Italy (IRB 2018_468). Informed consent was obtained from all subjects involved in the study. Patients’ anonymity has been guaranteed.

## 3. Results

### 3.1. Aided versus Unaided Hearing Thresholds

The patients’ AC pure-tone average (PTA) across 0.5, 1, 2, and 4 KHz was 66.9 dB HL (±16 dB HL) for the right ear and 66.7 dB HL (±10.4 dB) for the left ear. The air–bone gap (ABG) average at 0.25, 1, and 2 KHz was 37.1 dB HL (±9.7 dB HL) for the right ear and 37.3 dB HL (±10.4 dB HL) for the left ear.

**Figure 1:**
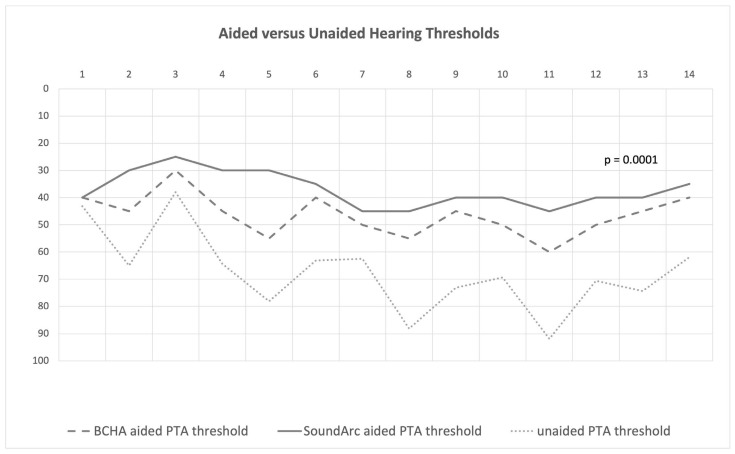
SoundArc, BCHA, and unaided differences in each patient. The cases (# ID) are reported on the horizontal axis, whereas the vertical axis describes the hearing thresholds (dB HL).

The mean BCHA-aided threshold was 46.4 ± 7.7 dB HL, and the mean SoundArc-aided threshold was 37.1 ± 6.4 dB HL, resulting in a significant functional gain for both the aided conditions versus the unaided condition (*p* = 0.0001) (Figure 1). Only two patients (#1 and #3) did not show a significant improvement; they were the only two patients with moderate conductive hearing loss and good BC thresholds (Table 1).

**Figure 2:**
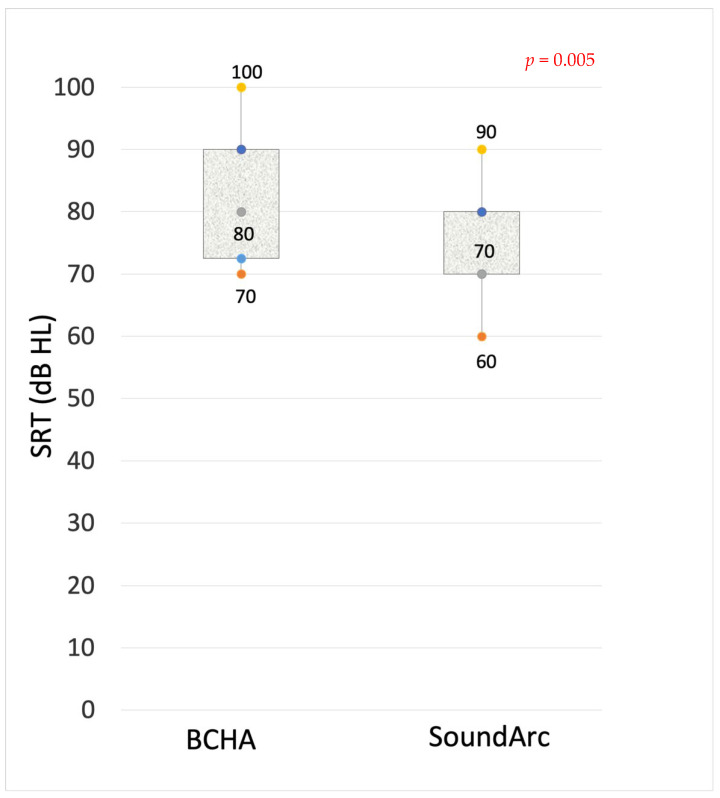
The maximum intelligibility thresholds with BCHAs and SoundArc in quiet. The reported boxplot dots are the quartiles: the minimum value, the first quartile (Q1, 25° percentile), the median (Q2, 50° percentile), the third quartile (Q3, 75° percentile), and the maximum value.

**Figure 3:**
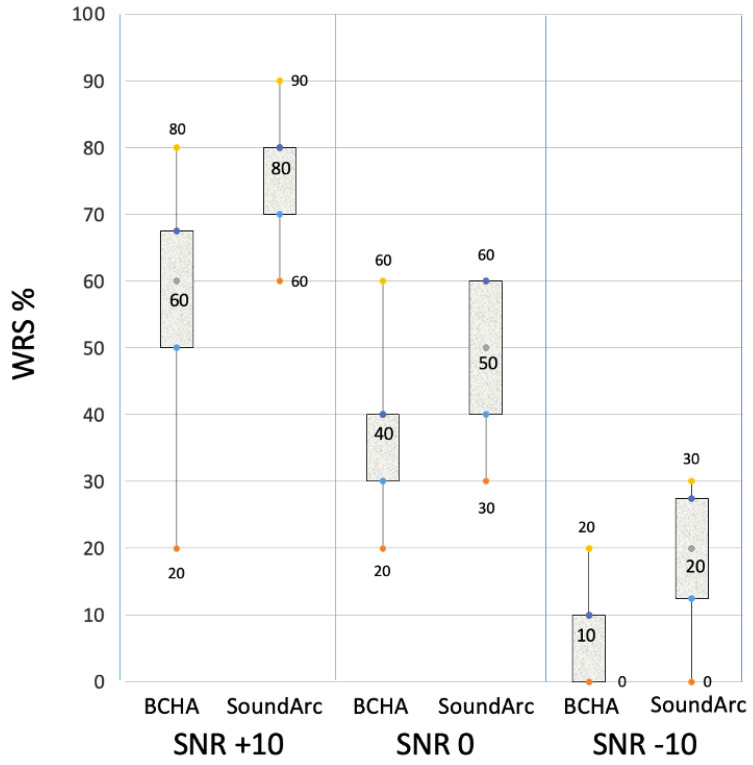
Word repetition scores with BCHAs and SoundArc. The reported boxplot dots are the quartiles: the minimum value, the first quartile (Q1, 25° percentile), the median (Q2, 50° percentile), the third quartile (Q3, 75° percentile), and the maximum value.

Speech tests in quiet and noise. Raw data for the single patients are reported in Table 2. Speech perception outcomes expressed as speech reception thresholds (SRTs) are reported in Figure 2. With SoundArc, patients achieved slightly better speech perception in quiet. The Wilcoxon test provided a statistically significant difference between the two groups; in particular, the SRT of 100% correct answers was at 80 and 70 dB HL, respectively (*p* = 0.005). As reported in Figure 3, more consistent differences between BCHAs and SoundArc were found in noise: the WRS in noise was improved with SoundArc in all the tested conditions: +19.3 at SNR +10 dB, *p* = 0.002; +12.1 at SNR 0 dB, *p* = 0.006; and +11.4 at SNR −10 dB, *p* = 0.002.

### 3.2. Subjective Outcomes/Questionnaires

The SSQ scores were remarkably better for SoundArc, with improvements at a significant level for all three sub-domains, especially for “speech” (Table 3). Similarly, all subjects reported significant improvements in VAS scores for two of the three items (+7.6 ± 1 for comfort, +3.0 ± 1 for weight), while the subjective judgment of aesthetic appeal was essentially unchanged (+1.1 ± 1.3).

Conversely, the patients did not report any significant subjective benefits at IOI-HA (4.0 ± 1.0 for BCHAs; 4.0 ± 1.0 for SoundArc, *p* = n.s.).

The HHIA/HHIE scores revealed the notable absence of reduced participation restriction, with no statistically significant differences between SoundArc and BCHAs in any of the measures. In agreement with this, all patients reported low levels of listening fatigue, as assessed by FIS scores (ranging from 0 to 3), in both aided conditions.

## 4. Discussion

The present study demonstrates that, despite the fact that patients were experienced users of their BCHAs, having used them for many years, SoundArc BCHAs provided better speech perception performance, especially in a noisy background, and improved SSQ scores, despite the brevity of the trial period. Surprisingly, all patients reported their appreciation in terms of the comfort of use of this rigid bow. On the other hand, the results of other subjective reports of benefits (HHIA/HHIE, FIS) were inconsistent. These reports might be more influenced by a more prolonged use of hearing devices.

As far as we know, this is the first report comparing functional gains between conventional BCHAs on eyeglasses and SoundArc in patients with chronic conductive/mixed hearing loss who are used to the old, traditional system in order to understand whether it is reasonable to propose SoundArc as an alternative or not.

The first description of a sound conduction tool was reported by Girolamo Cardano, who simply explained that it was possible to “…*hear distant sounds, voices and words that he could not hear under normal circumstances*…” using a rod or the shaft of a spear held between one’s teeth [30]. At the beginning of the 20th century, the development of the carbon microphone speaker allowed the construction of a BC vibrator placed on the mastoid area, notably supported by eyeglasses since the 1950s. BCHAs, usually mounted on specially designed eyeglasses, have been the traditional and easiest solution for conductive hearing loss. However, their appreciation and diffusion have been limited by inefficient coupling with the temporal bone, by partially unsatisfactory results, and also for aesthetic reasons. The literature from the last 40 years on the indications for BCHIs is extensive, ranging from external and middle ear congenital malformations to failures of middle ear surgery for COM or otosclerosis [1]. In response to the demand for a non-surgical BC solution, two different options were developed in order to benefit from BCHI sound processors: headbands/softbands and adhesive fixation. These solutions are often used for temporary preoperative trials, and occasionally also for permanent use, especially in young children with congenital conductive hearing loss, such as external ear atresia and ossicular fixation, or until skull growth allows the implantation of the fixture [31]. A comparison between SoundArc and a softband was reported in a previous study by Gawliczek et al. in 2018 [18]. The authors reported considerable improvements in hearing and speech understanding in subjects with a simulated, purely conductive, bilateral hearing loss, but no significant difference between the two wearing options was found, probably due to the use of the same device and to the similar force measurements in the two coupling methods. As a matter of fact, so far, all these solutions have seen limited success, especially in older children and in adults, mainly due to their low aesthetic appeal [32,33,34], but also for the significant sound attenuation produced by the skin, which can reach 15 dB HL at 3 KHz [6,31,35]. These limitations are also present in other solutions, such as the Adhear.

This study had the following main limitations: first, the paucity of patients enrolled does not allow us to draw definitive conclusions; second, the short duration of the trial period with SoundArc compared to the long-term acquaintance with BCHAs (>3 years) might skew the statistical analysis; and lastly, the study enrolled only elderly people, and future studies may be necessary for younger people’s evaluation. The lack of a difference in subjective reports against a significant evidenced benefit suggests that some results might be different after a longer use of SoundArc. Therefore, the outcomes of the present study must be considered preliminary. Furthermore, the major cost of non-wearable solutions of passive BCHIs compared to BCHAs might have influenced subjective reports. The main benefit derived from SoundArc, in the selected sample of patients in the present study, appears to be an advantage in speech perception under unfavorable listening conditions, especially for those with greater hearing loss. A possible explanation is presumably related to the better coupling of SoundArc in comparison to BCHAs mounted on eyeglasses, as a better coupling effect of SoundArc compared to a headband has already been reported [18]. These mechanical aspects will be the object of further study.

In conclusion, SoundArc represents a viable alternative to BCHAs mounted on eyeglasses. It should be included in the list of non-surgical wearable options for patients not suitable for or refusing implantable devices (BCHIs), as its clinical functional outcomes surpass those of traditional BCHAs.

## Figures and Tables

**Table 1:** Patients’ details.

ID	Sex	Age (years)	Etiology of HL	Prior Surgery	Conductive/Mixed (C/M)	Years of Eyeglass BCHA Use	PTA4 AC (dB HL)	ABG (dB HL)
1	M	75	Chronic otitis media, radical surgery	yes	C	11	43	28
2	F	80	Unknown	no	M	11	65	44
3	F	70	Chronic otitis media	no	C	16	38	25
4	F	74	Otosclerosis	no	M	3	64	48
5	F	79	Chronic otitis media	no	M	3	78	49
6	F	83	Chronic otitis media	no	M	10	63	34
7	M	68	Chronic otitis media, myringoossiculoplasty	yes	C	9	63	32
8	M	81	Chronic otitis media	no	M	8	88	45
9	F	78	Otosclerosis, otitis media	no	M	9	73	39
10	M	79	Chronic otitis media, myringoplasty	yes	M	14	69	39
11	M	88	Chronic otitis media	no	M	17	92	39
12	F	77	Chronic otitis media, narrow external auditory canal	no	M	6	71	35
13	F	76	Chronic otitis media	no	M	8	74	33
14	M	81	Chronic otitis media	no	M	12	62	32
Overall mean ± SD	8F; 6M	77.8 ± 5.1	-	-		9.8 ± 4.2	67.4 ± 14.7	37.3 ± 7.3

**Table 2:** Speech test outcomes in quiet and in noise.

	Speech in Quiet		Speech in Noise (% of Correct Responses—WRS at 60 dB HL)					
	SRT dB HL		SNR +10		SNR 0		SNR −10	
ID	BCHA	SoundArc	BCHA	SoundArc	BCHA	SoundArc	BCHA	SoundArc
1	70	60	20	70	20	30	0	0
2	70	60	60	90	40	60	10	30
3	100	80	50	70	30	50	0	10
4	100	70	60	80	30	60	10	30
5	90	80	60	60	40	40	0	20
6	80	80	70	80	60	60	20	30
7	80	70	30	80	20	40	0	10
8	90	80	50	70	30	40	0	10
9	70	70	80	80	50	50	20	20
10	80	70	50	80	40	60	10	20
11	100	90	60	60	40	40	10	20
12	80	70	60	80	30	50	0	20
13	80	80	70	70	50	50	20	30
14	70	70	70	90	40	60	10	20
Overall mean ± SD	82.9 ± 11.4	73.6 ± 8.4	56.4 ± 16	75.7 ± 9.4	37.1 ± 11.4	49.3 ± 10.0	7.9 ± 8.0	19.3 ± 9.2
Wilcoxon paired test (*p*-value)	*p* = 0.005		*p* = 0.005		*p* = 0.006		*p* = 0.002	

**Table 3:** SSQ questionnaire data.

	SSQ					
	Speech		Spatial		Qualities	
ID	BCHA	SoundArc	BCHA	SoundArc	BCHA	SoundArc
1	3	7.5	3	7	3	8
2	6	7	6	7.5	5.5	7
3	5	7.5	5	8	4.5	7.5
4	4.5	8	4.5	7.5	5	7.5
5	6	6	6	6.5	6	6
6	6	6.5	6.5	6.5	6	7
7	3	8	3	7.5	3.5	8
8	6	7.5	6	7	5.5	7
9	7	7	7	7	7.5	7.5
10	4.5	7	4.5	7	4	6.5
11	5	5	4	5	5	5
12	5.5	8	5	7.5	5	8
13	7	7	7	7	7	7.5
14	7	8.5	6.5	8	7	8.5
Overall mean ± SD	5.5 ± 1.4	7.0 ± 1.0	5.2 ± 1.5	7.0 ± 0.9	5.3 ± 1.3	7.0 ± 1.1
Wilcoxon paired test (*p*-value)	0.005		0.003		0.003	

## Data Availability

Data are available on request.
